# Socioeconomic Status in Pregnant Women and Sleep Quality During Pregnancy

**DOI:** 10.7759/cureus.6183

**Published:** 2019-11-18

**Authors:** Livier J Silva-perez, Natalia Gonzalez-Cardenas, Sara Surani, FA Etindele Sosso, Salim R Surani

**Affiliations:** 1 Medicine, Autonomous University of Baja California, Tijuana, MEX; 2 Medicine, Universidad Central de Venezuela, Caracas, VEN; 3 Global Health, Harvard University, Cambridge, USA; 4 Center of Advanced Research in Sleep Medicine, Hopital du Sacre-Coeur de Montreal, Montreal, CAN; 5 Internal Medicine, Texas A&M Health Science Center, Temple, USA

**Keywords:** poor quality of sleep, pregnancy, low socioeconomic status, sleep in pregnancy

## Abstract

This review focuses on factors contributing to sleep quality among pregnant women with low socioeconomic statuses during the third trimester of their pregnancy. Electronic searches were conducted, following the Preferred Reporting Items for Systematic Reviews and Meta-Analysis (PRISMA) guidelines. We searched for published, peer reviewed, English language primary research articles using electronic databases including PubMed, EMBASE, Ovid, MEDLINE and Google Scholar ending June 2019. All references were reviewed manually and independently by authors. After applying the inclusion criteria, 56 articles were selected; 38 of which are full-text and included in this review. All articles related to the analysis of poor sleep quality among uncomplicated pregnant women were included. Pregnant women with a specific pathology were excluded.

We found poor sleep quality among pregnant women is correlated with low socioeconomic levels. Pregnant women with lower incomes tend to have inadequate diets, which further complicates the health of the mother and the baby. External factors including low income, poor quality of life and poor diet tend to increase the possibility of future health complications in both mother and child, and can result in complications such as preterm labor, low birth weight, preeclampsia, perinatal death, and spontaneous abortion.

## Introduction and background

Sleep is a natural state of altered consciousness where the nervous system is relatively inactive. Sleep quality and duration are important for a number of physiological functions [1]. During pregnancy, combination of hormonal effects, mechanical effects due to enlarging uterus and the circulatory changes causes significant alteration in the sleep quality. Study by Mindell et al. in their survey of 2427 pregnant women showed that 76% of women reported poor sleep quality and 38% had insufficient sleep [2]. According to National Sleep Foundation, in order to achieve good sleep quality, an individual must sleep at least 85% of the time in bed, should fall asleep within the first 30 minutes and should not wake up, no more than once during the night. If the individual wakes up, he or she should be able to fall back to sleep within 20 minutes [3].

During pregnancy there is also alteration in the normal sleep with less slow wave sleep and increase sleep fragmentation [4]. In addition, alteration in sleep can have significant health and cardiovascular events. The association between pregnancy and sleep disturbances varies from insomnia, nighttime awakenings, parasomnias, and snoring. These sleep alterations are a consequence of hormonal and physical changes related to pregnancy. Moreover, poor sleep quality from physiological or pathological causes seems to be more prevalent during the third trimester of pregnancies [5, 6]. During pregnancy, many physiological changes occur to achieve a homeostatic equilibrium for the benefit of the fetus. Particularly, the sleep adaptive modifications are present in the respiratory and cardiovascular systems, which are constantly responding to a myriad of hormonal events [7]. For example, increases in beta-human chorionic gonadotropin (HCG) and progesterone change sleep patterns. While estrogen shortens the duration of rapid eye movement (REM)-sleep, progesterone stimulates sleep by balancing the duration of the REM sleep stage and non-REM (NREM) stage for the health of the mother [8]. Additionally, estrogen influences an increase of secretions, vascularity, and edema in the respiratory tract. Moreover, a decrease of functional residual capacity and the dimension of airways results in shortness of breath. Rising levels of cortisol influence the activation of sympathetic tones, resulting in low vascular peripheral resistance and higher maternal heart rates. In summary, as time passes, these changes contribute to alterations of pre-pregnancy sleep patterns including shorter sleep durations, night awakenings, snoring, and gasping [9].

During pregnancy, alterations of sleep patterns are common. Prevalence varies widely from 30 to 78% across studies [10-12]. Beebe et al. characterized the symptoms experienced in late pregnancy, and reported that 68% of women had sleep disturbances and scored these disturbances highest for severity, frequency and distress [12]. Similarly, Hutchison et al. found that women reported both decrease in hours of sleep (8.1 to 7.5 h) and poor quality of sleep (up to 61%) during their third trimesters [9]. Likewise, a survey of 2427 women reported that pregnant women had poor quality of sleep (76%), insomnia (57%), night awakening (100%) and sleep-breath alterations (19%) from the beginning until the end of their pregnancies [2].

In a case-control study by Sut et al. from Turkey, sleep quality among pregnant women was poor than in their non-pregnant peers [13]. Socioeconomic status is a concept which considers the level of education, income, and occupation of the individual. There is a considerable evidence that low socioeconomic status is linked to obstetrical complications such as preterm deliveries, high rate of caesarean sections and third trimester hemorrhages [14].

Prior studies suggest that low-income pregnant women were at high risk of poor quality of sleep when compared with high-income pregnant women [15,16]. The spectrum of sleep disorders in developed countries is associated with poor maternal-fetal outcome. It seems that health care providers may overlook sleep disorders as a common complaint during gestation and shortly after delivery.

Understanding sleep patterns during pregnancy may have significant impacts on gestational outcomes [17]. During pregnancy, poor quality of sleep (PQOS) and sleep disorders have been associated with hypertensive states like preeclampsia (Preeclampsia can occur as early as 20 weeks of gestation, if one is at risk than symptoms usually occurs around 34th week) [18,19]. Short sleep duration has also been linked to the development of gestational diabetes, high risk of preterm labor, and cesarean delivery [20,21]. Furthermore, Dolatian et al. reports that lower socioeconomic status and high levels of stress have shown correlation with lower birth-weights [22].

Despite data suggesting sleep disturbances among pregnant patients, the influence of socioeconomic status during last months of pregnancy is not clearly understood [21, 23-25]. This mini review focuses on presenting factors contributing to poor sleep quality among pregnant patients with lower income and socioeconomic statuses, especially during their third trimesters.

## Review

Methods

**Figure 1:**
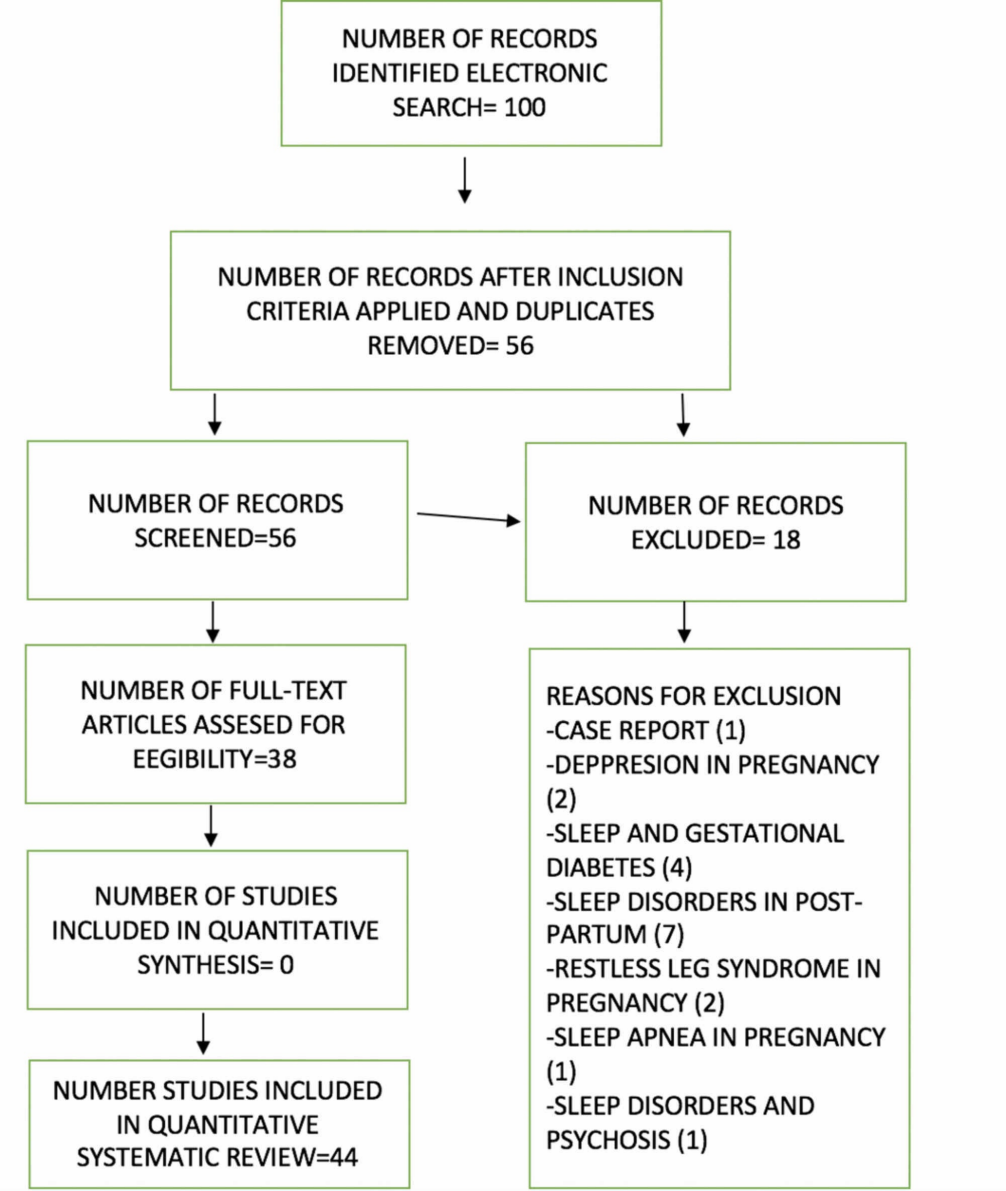
PRISMA flow chart. PRISMA: Preferred Reporting Items for Systematic Reviews and Meta-Analysis

We conducted an electronic-based search following the Preferred Reporting Items for Systematic Reviews and Meta-Analysis (PRISMA) guidelines. We searched for published, peer reviewed, English language primary research articles using electronic databases as PubMed, EMBASE, Ovid, MEDLINE, and Google Scholar. The following medical subject heading (Mesh) terms, keywords, and their combinations were used: “pregnancy”, “low socioeconomic status”, “poor quality of sleep”. The search was limited to studies published up until June 2019. We manually searched the reference lists of identified studies. We included all original articles as well as systematic reviews. Three authors (LS, NG, SS) assessed each article independently. Our search, after excluding duplicates, non-English articles, non-related citations to key words, and unavailable full text was filtered to 38 articles. These articles included randomized studies, cross sectional observational studies, quasi experimental studies and case control studies. One hundred articles in total were reviewed (See Figure 1).

Discussion

The sleep time required for a pregnant woman is approximately 7.5 hours or more [26]. The Pittsburg Classification Sleep Quality Index was the most commonly used instrument to measure sleep quality [27]. These questions focus on assessing the quality of sleep based on duration, disturbances, latency, daytime dysfunction, efficiency, and quality among other characteristics.

There are several factors that affect sleep quality during pregnancy; socioeconomic levels being the only indicator that also affects the quality of life of the pregnant woman. Additionally, when sleep is interrupted by physical-hormonal-psychological changes, one can see poor outcomes in both the woman and the newborn [16].

According to the American Community Survey 2013-2017, the average household income in the United States was $57,652 [28]. A household income less than $50,000 per year is considered a low socioeconomic status. It is well known that Medicaid is an important health care service for pregnant women with lower incomes and that Medicaid is responsible for health coverage of approximately 50% of all births in the United States [2]. Moreover, a recent study reported that premature births and poor health are often observed in women who receive Medicaid [29]. To further complicate matters, pregnant women with low income have a higher probability of presenting long-term conditions during the gestation process [30].

Sleeping through the night during pregnancy is crucial for the health of both the mother and the fetus. At the same time, it is challenging to get sufficient sleep because of interruption by socioeconomic, physical, and psychological factors. According to the Sedov et al., in the third trimester of gestation, insomnia is present more often due to muscle-skeletal pain, overweight physique, restless legs, reflux, non-comfortable positions and snoring [31]. Night awakenings during the third trimester are more frequent due to an uncomfortable muscle-skeletal pain [32]. In a cross-sectional study in pregnant women, back pain was present in 55% to 75% of pregnant women during pregnancy. Mirroring these observations, Yikar and Nazik also found that the most common complaints during pregnancy are seen in second and third trimester. Furthermore, the symptoms reported during the second and third trimester were fatigue, exhaustion, restless legs, backache, nocturia, depression, and anxiety episodes [33].

Dolatian et al. found that women who sleep less than 8 hours have a higher risk of cesarean-section deliveries, preterm labor, and decrease in baby size when compared with their gestational age increases by 2.2 fold [34]. Another study showed that pregnant women with Medicaid have a higher probability of C-section or preterm deliveries [35]. Moreover, Bruce also showed that pregnant women with low socioeconomic status or financial insecurity demonstrate a tendency to have a poor quality of life which affects the health of pregnant women [36]. Table 1 illustrates the findings of some of the key studies suggesting sleep disturbances during pregnancy and also the effects of socioeconomic status.

**Table 1:** Studies suggesting sleep disorder during pregnancy.

Study	Type of study	Subjects	Outcomes
Romero and Badr, 2014 [6]	Systematic review	-Two systematic reviews and meta-analyses provide evidence of an association between sleep-disordered breathing and adverse pregnancy outcome.	-Sleep disorders may be associated with complications of pregnancy, such as gestational diabetes, gestational hypertension, preeclampsia, preterm birth, stillbirth.
Facco et al., 2010 [10]	Prospective, cohort	-Nulliparous women - 6 and 20 weeks of gestation - who completed a baseline sleep survey at enrollment with follow-up in the third trimester. -Berlin Questionnaire for Sleep Disordered Breathing; Epworth Sleepiness Scale; National Institutes of Health; International Restless Legs Syndrome Question Set; Women's Health Initiative Insomnia Rating Scale; Pittsburgh Sleep Quality Index	Sleep disturbances are prevalent among healthy nulliparous women and increase significantly during pregnancy.
Mindell et al., 2015 [2]	Prospective, cohort	-2427 women -Pittsburgh Sleep Quality Index (PSQI); Epworth Sleepiness Scale; Vitality scale of the Short Form 36 Health Survey (SF-36); Insomnia Severity Index (ISI); Berlin Questionnaire; International Restless Legs Syndrome (IRLS) question set short version of the Pregnancy Symptoms Inventory (PSI).	Women experience significant sleep disruption and inadequate sleep throughout pregnancy
Sut et al., 2016 [13]	Cross sectional survey	-492 women (292 pregnant and 200 non-pregnant healthy control); Sociodemographic; Pittsburgh Sleep Quality Index (PSQI); European Quality of Life-5 Dimensions (EQ-5D)	Sleep quality and health-related quality of life of pregnant women were worse than those of non-pregnant healthy controls.
Kim et al., 2018 [14]	Prospective observational	From National Health Insurance database in Korea, we selected women who gave birth between January 1, 2010 and December 31, 2010. As an indicator reflecting socioeconomic status (SES), we classified subjects: MA (“low” SES), NHI beneficiary (“middle/high” SES).	Women in the MA group showed high rates of miscarriage, caesarean, preeclampsia, preterm delivery, and hemorrhage than those in the NHI group.
Okun et al., 2014 [16]	Prospective, longitudinal	One hundred seventy pregnant women at 10-20 weeks gestation; socioeconomic status was defined by self‐reported annual household income; linear regression analyses independent associations of SES on sleep after adjusting for age, race, parity, marital status, body mass index (BMI), perceived stress, depressive symptoms, and financial strain.	Low SES was associated with poor sleep quality and fragmented sleep.
Xu et al., 2017 [15]	Cross-sectional survey	Pregnant women between June and August 2015 from 16 hospitals in five provinces in China. A total of 2345 pregnant women - 18 years and older - were surveyed.	High-risk groups of poor sleep quality; women of non-Han nationality; low income level; third trimester of pregnancy; insufficient sleep duration.
Li et al., 2017 [21]	Prospective	-688 healthy women with singleton pregnancy were selected from three hospitals in Chengdu, China (2013-2014); self-report questionnaires - the sleep quantity and quality; exercise habits in 12, 16, 24, 28, 32, and 36 weeks; type of delivery; gestational age; neonates weight	Sleep disturbances are associated with an increased risk of cesarean delivery and preterm birth in pregnancy.
Nguyen et al., 2018 [37]	Prospective cohort	Eligible pregnant women were recruited from the cities of Vietnam, namely, Hanoi, Hai Phong, and Ho Chi Minh City between August 2015 and July 2016; interview at 24–28 weeks of gestation (lifestyle, dietary intake, physical activity, smoking, socio-demographics, and medical history).	They compared pregnant women with high and low incomes and found that women with medium and low income tend to have a deficiency in micronutrients intake compared to women with high income, and this is one predictor of future fetal poor outcomes because the micronutrients are involved in the metabolism of the fetus, growth, development, and function of the organs, and late chronic diseases.

Pregnant women with low socio-economic status tend to receive inadequate nutrients [37]. Another article found how women with lower incomes have poorer diets and consume higher levels of saturated fatty, carbohydrates, soft beverages, or food without adequate nutrients. An investigation shows how high fatty food consumption, especially in women with overweight physiques, increases the possibility to develop obesity, gestational diabetes, abortion, preeclampsia. There is also a high possibility that the baby will develop diabetes mellitus type 2 in their adulthood [38]. The European Food Safety Authority 2014 recommends that pregnant women have an additional calorie intake in every trimester. For example, the 1st trimester should be 70 kcal per day, 2nd trimester should be 260 to 500 kcal per day, and 3rd trimester should be 500 kcal per day, to maintain a balance due to increase in demands during pregnancy [39].

According to the World Health Organization, in order to improve maternal and fetal wellbeing, a balanced intake during gestation must contain the following nutrients: green leafy vegetables, protein (fish, salmon, meat), cereal, beans, and nuts [40]. The salmon contains an elevated quantity of Omega 3 fatty acid, which plays an important role in the brain and retina formation of the fetus and also reduces the possibility of preterm delivery [41]. Iodine is a micronutrient required for metabolic and hormonal functions during pregnancy. Decreased intake may cause abortion, brain damage in the fetus, or mortality in the perinatal period [42]. Calcium is involved in fetus development, important for normal birth weight, decreases the risk of premature delivery, and controls blood pressure during pregnancy [43]. Folic acid consumption is necessary for preventing neuro-tube congenital defects or heart disease, and also helps with adequate placenta formation [44].

RDA Italy recommends regular intake of these micronutrients for positive benefits during the gestational process: Omega 3 (250 mg), Iodine (250 mg), Calcium (1200 mg), Folic acid (600 mg), Vitamin D (15 mg). All these nutrients optimize the requirements during pregnancy [39].

## Conclusions

In this review, we focused on how low socioeconomic status affects the quality of sleep in pregnant women during and after the third trimester of pregnancy. As in our discussion, we suggested that factors involved in the poor quality of sleep during pregnancy are related, and how low socioeconomic levels are associated with poor quality of life. The controlled trials of high quality though are lacking in these areas. A pregnant woman with a low income who tends to have an inadequate diet may have future health complications for both herself and her fetus. During pregnancy, the body undergoes physical, hormonal, and physiological changes that are magnified by external factors including low income, poor quality of life, and poor diet. These factors tend to increase the possibility of future health outcomes in both, mother and fetus and can result in preterm labor, low birth weight, preeclampsia, perinatal death, and spontaneous abortion. Further research is needed to evaluate the differences between high- and low-income groups in comparison to their non-pregnant peers. Clinicians should take advantage of assessing instruments of quality of sleep to identify pregnant women at risk of poor perinatal outcomes. Nutritionists should asses and identify pregnant women with high risk for future poor outcomes.
